# Differential rates of guideline-directed medical therapy modification in C-MIC and control patients: results from the C-MIC II trial

**DOI:** 10.1093/eschf/xvag220

**Published:** 2026-08-18

**Authors:** Marat Fudim, Tamara Kovacevic-Preradovic, Dragana N Kosevic, Sasko Jovev, Nermir Granov, Tanja Popov, Igor Rudez, Petar Vukovic, Velibor Ristic, Annette Holtdirk, Miodrag Peric, Faouzi Kallel, Javed Butler, Jesus Eduardo Rame, Stefan D Anker, Marija Zdravkovic

**Affiliations:** Department of Cardiology, Duke University, Durham, NC, USA; Faculty of Medicine, University of Banja Luka, Banja Luka, Bosnia and Herzegovina; Cardiovascular Institute Dedinje, Belgrade, Serbia; University Clinic of Cardiology, Skopje, North Macedonia; Clinical Center University of Sarajevo, Sarajevo, Bosnia and Herzegovina; Institute of Cardiovascular Diseases of Vojvodina, Vojvodina, Serbia; Department of Cardiac and Transplant Surgery, University Hospital Dubrava, Zagreb, Croatia; University Belgrade Medical School, Belgrade, Serbia; Cardiovascular Institute Dedinje, Belgrade, Serbia; RQM+, Frankfurt, Germany; Cardiovascular Institute Dedinje, Belgrade, Serbia; Berlin Heals, GmbH, Berlin, Germany; Baylor Scott and White Research Institute, Dallas, TX, USA; Department of Medicine, University of Mississippi Medical Center, Jackson, MS, USA; Bruce and Robbi Toll Heart and Vascular Institute, Thomas Jefferson University, Philadelphia, PA, USA; Deutsches Herzzentrum der Charité, Campus Virchow Klinikum, Berlin, Germany; Institute of Health Centre for Regenerative Therapies (BCRT), German Centre for Cardiovascular Research (DZHK) Partner Site Berlin, Charité Universitätsmedizin, Berlin, Germany; Department of Cardiology, University Hospital Medical Center Bezanijska Kosa, Belgrade, Serbia

**Keywords:** Cardio-microcurrent device, Electrical microcurrent, Heart failure

## Abstract

**Background:**

In the C-MIC II trial, C-MIC therapy improved outcomes in patients with heart failure (HF) with reduced ejection fraction (HFrEF). We evaluated whether differences in background HF medication adjustments influenced the observed benefits.

**Methods:**

Ambulatory patients with chronic non-ischaemic HFrEF receiving guideline-directed medical therapy (GDMT) were enrolled. The primary outcome was change in GDMT and diuretic intensity over 6 months. Treatment effects on left ventricular ejection fraction (LVEF), Kansas City Cardiomyopathy Questionnaire Overall Summary Score (KCCQ-OSS), and 6-minute walk distance (6MWD) were assessed by baseline sodium-glucose cotransporter-2 inhibitor (SGLT2i) use for consistency.

**Results:**

Among 65 patients, 25 (39%) had GDMT adjustments (C-MIC device: 44%; control 33%; *P* = .390). GDMT intensity increased in controls (*Δ* + 0.36) but decreased (*Δ* −0.16; *P* = .100) in C-MIC group. Diuretic intensity decreased in the C-MIC group (*Δ* −0.10) but increased in controls (*Δ* + 0.12; *P* = .200). When stratified by baseline SGLT2i use, C-MIC therapy induced consistent improvements in LVEF (SGLT2i: +6% [95% CI 3–9; *P* < .001] vs. without SGLT2i: +4% [95% CI 2–7; *P* = .003]), KCCQ-OSS (SGLT2i: +42 points [95% CI 26–58; *P* < .001] vs. without SGLT2i: +40 [95% CI 28–51; *P* < .001]) and 6MWD (SGLT2i: +150 metres [95% CI 88–212; *P* < .001] vs. without SGLT2i: +141 metres (95% CI 63–219; *P* < .001]).

**Conclusion:**

Medication intensity decreased in C-MIC-treated patients but increased in controls, driven primarily by escalation of diuretics, likely reflecting worsening HF. Consistent improvements in LVEF, 6MWD and KCCQ-OSS across SGLT2i subgroups suggest that the observed benefits are independent of background pharmacologic intensification.

## Graphical Abstract

**Graphical Abstract:**
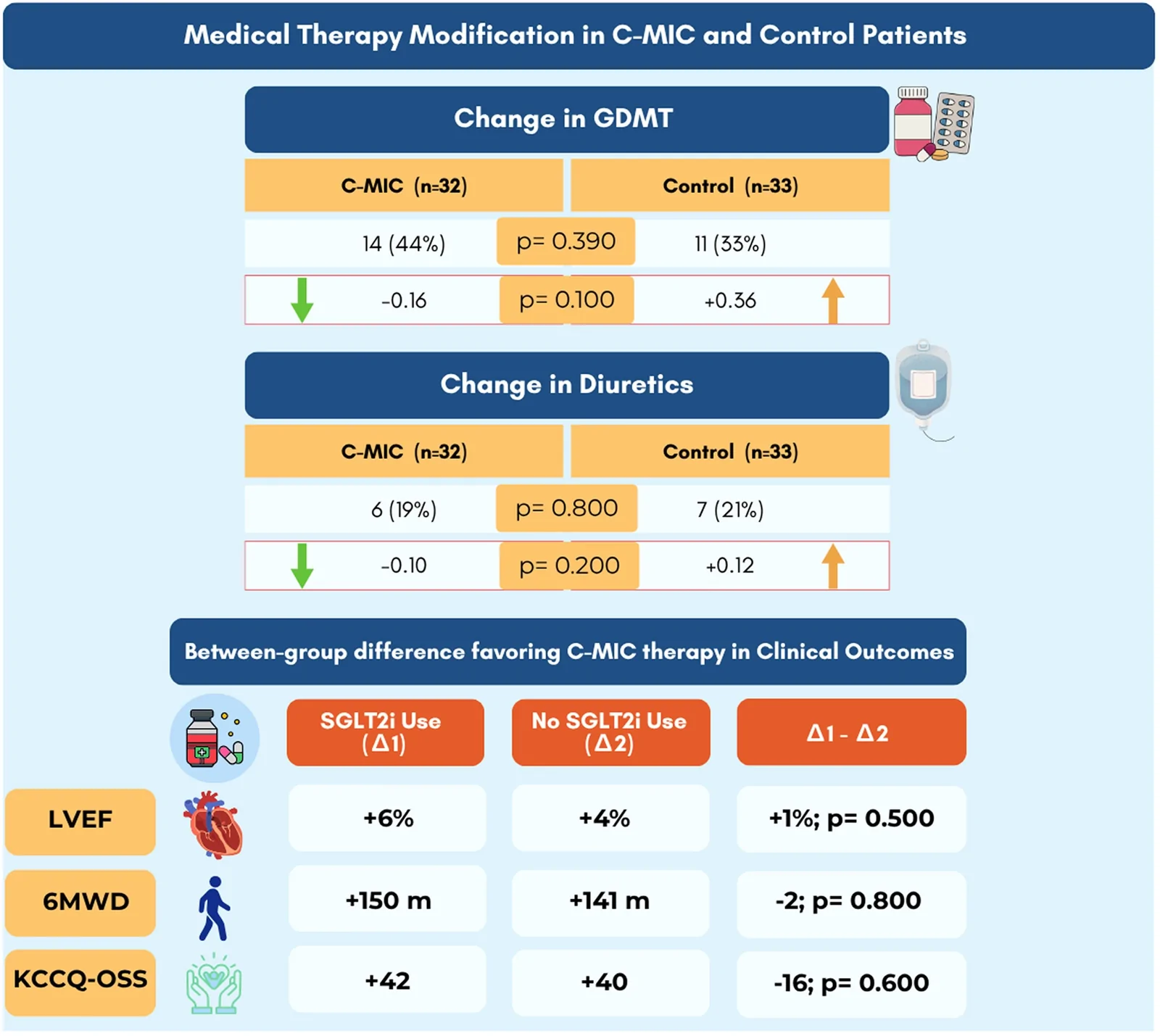
Differential changes in HF medication intensity in C-MIC-treated patients vs controls, and the influence of baseline SGLT2 inhibitor (SGLT2i) use on clinical outcomes. A cumulative medication score was calculated for each patient at baseline and at final follow-up, with the change in score (*Δ*) used to quantify longitudinal modifications in guideline-directed medical therapy (GDMT) and diuretic intensity. Initiation or discontinuation of a medication resulted in positive or negative points, respectively, while major dose escalations or reductions were assigned ±2 points and minor dose changes were assigned ±1 point.

## Introduction

Randomized clinical trials in heart failure (HF) are increasingly conducted in a therapeutic environment characterized by the rapid evolution of guideline-directed medical therapy (GDMT). As a result, interpretation of treatment effects, particularly in trials evaluating device-based or non-pharmacologic interventions, may be confounded by background medication changes occurring during follow-up. This issue is especially relevant in modest-sized randomized trials, where even subtle imbalances in medication initiation, titration, or timing may meaningfully influence functional, structural, or patient-reported outcomes.

Over the past decade, multiple landmark trials have established angiotensin receptor–neprilysin inhibitors (ARNI), mineralocorticoid receptor antagonists (MRA), and sodium–glucose cotransporter-2 inhibitors (SGLT2i) as foundational therapies for heart failure with reduced ejection fraction (HFrEF).^1–3^ In particular, Dapagliflozin and Prevention of Adverse Outcomes in Heart Failure (DAPA-HF) and Empagliflozin Outcome Trial in Patients with Chronic Heart Failure and a Reduced Ejection Fraction (EMPEROR-Reduced) demonstrated that SGLT2is significantly reduce HF hospitalization and cardiovascular mortality irrespective of diabetes status, with early separation of event curves suggesting rapid clinical benefit.^1,2^ Subsequent trials, including Empagliflozin in Patients Hospitalized With Acute Heart Failure Who Have Been Stabilized (EMPULSE) and Effect of Sotagliflozin on Cardiovascular Events in Patients with Type 2 Diabetes Post Worsening Heart Failure (SOLOIST-WHF), further showed that early initiation of SGLT2is, during or shortly after hospitalization, confers early improvements in symptoms, functional status, and overall clinical benefit.^4^

Mechanistic and imaging studies have suggested that SGLT2is may exert favourable effects on myocardial remodelling, including reductions in left ventricular volumes, improvements in diastolic function, and modulation of myocardial fibrosis, within relatively short timeframes.^5^ Given these rapid and multi-mechanistic effects, differential initiation or titration of SGLT2is during a clinical trial could plausibly confound observed changes in left ventricular ejection fraction (LVEF), exercise capacity, or health-status measures. Accordingly, contemporary HF trial methodology emphasizes the importance of systematically accounting for background GDMT changes when interpreting treatment effects, particularly in trials evaluating non-pharmacologic or device-based therapies.^6,7^

Cardiac microcurrent (C-MIC) therapy represents a novel, non-excitatory bioelectronic approach intended to influence myocardial cellular signalling and tissue biology. The first-in-human clinical experience with C-MIC therapy demonstrated that the device could be safely implanted and delivered in patients with chronic HFrEF and was associated with improvements in LVEF, functional status, and exercise capacity without evidence of proarrhythmic or adverse device-related effects.^8^ Extended follow-up of this cohort showed that these benefits were durable through 2 years, with sustained improvements in cardiac function and clinical status.^9^

Building on these early findings, the C-MIC II trial was designed as a prospective, randomized study to evaluate the safety and efficacy of C-MIC therapy in ambulatory patients with chronic HFrEF receiving contemporary GDMT. The primary analysis demonstrated significant improvements in LVEF and multiple secondary clinical endpoints with C-MIC therapy compared with GDMT alone.^10^ Given the known impact of GDMT optimization, particularly SGLT2i initiation, on HF outcomes, the present analysis was undertaken to assess whether differences in HF medication adjustments during the trial may have contributed to or confounded the observed benefits associated with C-MIC therapy.

## Methods

### Study design and population

C-MIC II was a prospective, randomized, open-label controlled clinical trial enrolling ambulatory patients with chronic HFrEF. Eligible patients were New York Heart Association (NYHA) class III–IV with a LVEF between 25% and 35% and receiving stable GDMT 3 months prior to enrolment, consistent with contemporary HF trial design recommendations.^7,10^ Stable GDMT was defined as no more than a 50% increase or decrease in dose. Participants were randomized in a 1:1 fashion to receive either C-MIC therapy in addition to GDMT or GDMT alone. The present analysis was conducted in the modified intention-to-treat (mITT) population, defined as all randomized patients who received the assigned intervention and had at least one post-baseline assessment.

### Assessment of guideline-directed medical therapy

To be eligible for participation, patients were required to be on stable GDMT for HF at baseline. Target doses were not mandated, as up-titration is frequently limited by worsening symptoms, hypotension, renal dysfunction, or hyperkalaemia. HF medications and dose adjustments were prospectively collected at baseline and throughout follow-up. Medications assessed included GDMT for HFrEF (beta-blockers; angiotensin-converting enzyme inhibitors [ACEis], angiotensin II receptor blockers [ARBs], or ARNIs; MRAs; SGLT2is) and loop diuretics.

A semiquantitative medication scoring system was used to characterize baseline therapy and longitudinal changes in GDMT and diuretic intensity in the C-MIC and control groups. Each core GDMT drug class (ACE inhibitor/ARB/ARNI, beta-blocker, and MRA) was initially assigned 3 points, yielding a maximum score of 9. Following the incorporation of SGLT2is into guideline-directed therapy in 2021, the maximum possible score increased to 12. Diuretics were assessed separately and initially assigned 3 points.

Medication changes were scored based on both direction and magnitude. Initiation or discontinuation of a medication resulted in positive or negative points, respectively, with greater weighting assigned to earlier changes during follow-up. Dose adjustments were also captured, with major increases or decreases assigned ±2 points and minor changes assigned ±1 point. Medication changes occurring at the end of the study period were not scored.

A cumulative HF medication score was calculated for each patient at baseline and at final follow-up, and the change in score (*Δ*) was used to quantify longitudinal modifications in GDMT and diuretic intensity. The purpose of this score was to provide a standardized measure of changes in background pharmacologic therapy during follow-up. This approach is conceptually aligned with prior efforts to operationalize medication intensity in HF trials and registries.^7,11^

### Outcomes and subgroup analyses

The primary outcome of this analysis was the change in GDMT and diuretic intensity over the course of the study, assessed both as a continuous medication score and as the proportion of patients experiencing any medication adjustment.

Given robust evidence that SGLT2is exert early effects on symptoms, exercise capacity, and cardiac remodelling,^1–6^ treatment effects on LVEF, Kansas City Cardiomyopathy Questionnaire Overall Summary Score (KCCQ-OSS), and 6-minute walk distance (6MWD) were examined by baseline SGLT2i use to assess consistency.

### Statistical analysis

Baseline characteristics and medication use were summarized descriptively. Mean changes in medication scores were compared between groups. Categorical variables were compared using chi-square or Fisher’s exact tests, as appropriate. Treatment effects on clinical endpoints were estimated with corresponding 95% confidence intervals (CIs).

Changes in medication scores were evaluated separately for GDMT and diuretics. To assess the potential influence of background SGLT2i therapy, changes in clinical endpoints were evaluated separately in patients receiving SGLT2is at baseline and those not receiving SGLT2is. Formal tests for treatment-by–SGLT2i interaction were conducted to explore heterogeneity of treatment effects. All analyses were exploratory and hypothesis-generating. Missing efficacy data were not imputed and analyses were performed using available observed data. A two-sided *P*-value <.05 was considered statistically significant, and no adjustment was made for multiple comparisons.

## Results

### Study population

Of the 70 randomized patients, three patients in the device group withdrew before device implantation, and two patients in the control group withdrew after randomization. Sixty-five patients were included in the mITT population. Six-month follow-up data were available for all patients except for one patient in the control group who was lost to follow-up. At baseline, all participants had symptomatic chronic HFrEF treated with GDMT, with 28 patients (43%) receiving SGLT2i therapy. Two patients in the device group initiated SGLT2i therapy 14 days after randomization. The mean age of all participants was 60 ± 10 years, and 71% were men. The mean baseline LVEF was 31 ± 3% in the device group with SGLT2i use and 30 ± 4% among those not receiving SGLT2i therapy. All patients were NYHA class III, with the exception of one patient in the device group. At baseline, 13% of the participants in the device group had type 2 diabetes, 75% had hypertension, 9% had lung disease, and 9% had chronic kidney disease compared with 21%, 67%, 24%, and 0% of the participants in the control group, respectively. Baseline demographic and clinical characteristics, including GDMT and diuretic use and dosing, were similar between the C-MIC and control groups (*Table 1*). Serious adverse events were reported in six patients (5 in the device group vs. 1 in the control group). The rate of all hospitalizations was higher in the device group (risk difference: 12%, 95% CI −2 to 26).

**Table 1:** Baseline characteristics by treatment assignment and SGLT2i status

Characteristic	C-MIC		Control	
	On SGLT2i (*n* = 14)^a^	Off SGLT2i (*n* = 18)	On SGLT2i (*n* = 14)	Off SGLT2i (*n* = 19)
**Demographics**				
Age, years	56 ± 9	60 ± 10	62 ± 9	62 ± 10
Male sex, *n* (%)	11 (79)	12 (67)	9 (64)	14 (74)
Body mass index, kg/m^2^	30 ± 3	28 ± 5	31 ± 3	29 ± 3
**Heart failure characteristics**				
NYHA class III, *n* (%)	13 (93)	18 (100)	14 (100)	19 (100)
NYHA class IV, *n* (%)	1 (7)	—	—	—
LVEF, %	31 ± 3	30 ± 4	30 ± 3	29 ± 3
Duration of HF, years	2 ± 1	3 ± 2	3 ± 1	3 ± 2
**Vital signs**				
SBP, mmHg	122 ± 8	122 ± 13	119 ± 13	123 ± 14
DBP, mmHg	78 ± 7	80 ± 10	77 ± 8	77 ± 9
Heart rate, bpm	73 ± 15	77 ± 16	72 ± 17	77 ± 13
**Laboratory values**				
NT-proBNP, pg/ml, median (IQR)	551 (231, 730)	959 (270, 2517)	965 (365, 1423)	1049 (440, 2185)
eGFR, ml/min/1.73 m^2^	83 ± 33	77 ± 18	70 ± 20	86 ± 22
Haemoglobin, g/dl	148 ± 12	136 ± 13	151 ± 11	146 ± 12
**Functional status**				
6-minute walk distance, m	304 ± 53	303 ± 71	281 ± 55	278 ± 63
KCCQ-OSS	45 ± 21	46 ± 20	40 ± 20	40 ± 29
**Baseline HF medications**				
Beta-blocker, *n* (%)	13 (93)	18 (100)	14 (100)	18 (95)
ACEi/ARB/ARNI, *n* (%)	14 (100)	17 (94)	14 (100)	18 (95)
MRA, *n* (%)	13 (93)	16 (89)	14 (100)	19 (100)
Loop diuretic, *n* (%)	13 (93)	18 (100)	14 (100)	19 (100)

Data are expressed as mean ± standard deviation, unless otherwise indicated.

6MWD, 6-min walk distance; ACEi, angiotensin-converting enzyme inhibitor; ARB, angiotensin II receptor blocker; ARNI, angiotensin receptor-neprilysin inhibitor; C-MIC, cardio-microcurrent device; DBP, diastolic blood pressure; eGFR, estimated glomerular filtration rate; HF, heart failure; IQR, interquartile range; KCCQ, Kansas City Cardiomyopathy Questionnaire; LVEF, left ventricular ejection fraction; MRA, mineralocorticoid receptor antagonist; NT-proBNP, N-terminal pro-B-type natriuretic peptide; NYHA, New York Heart Association; SBP, systolic blood pressure; SGLT2i, sodium-glucose cotransporter-2 inhibitor.

^a^Two patients in the device group initiated SGLT2i therapy 14 days after randomization.

### HF medication adjustments

Overall, GDMT adjustments occurred in 25 patients (39%) during follow-up, occurring more frequently in the C-MIC group (44%) than the control group (33%). The likelihood of any medication change did not differ significantly between treatment arms (*P* = .390). However, medication intensity evolved differently over time. Mean GDMT scores decreased in the C-MIC group (*Δ* −0.16), whereas medication intensity increased in the control group (*Δ* + 0.36; *P* = .100), reflecting more frequent or larger dose escalations (*Table 2*).

**Table 2:** Overall change in GDMT during the study period

	C-MIC (*n* = 32)	Control (*n* = 33)	*P*-value (T-test)
Average baseline medication score (points)	9.66 ± 1.82	10.00 ± 1.76	.44
Average 6-month medication score (points)	9.50 ± 2.08	10.36 ± 1.92	.08
Overall change (points)	−0.16 ± 1.23	+0.36 ± 1.30	.1
Proportion of patients with change, ***n*** (%)	14 (44)	11 (33)	.39^a^

C-MIC, cardio-microcurrent device.

^a^Chi-square test.

Diuretics were adjusted in 13 patients (20%), with similar frequencies in the C-MIC group (19%) and the control group (21%; *P* = .800). However, diuretic intensity decreased in the C-MIC group (*Δ* −0.10) but increased in the control group (*Δ* + 0.12; *P* = .200) (*Table 3*).

**Table 3:** Overall change in diuretics during the study period

	C-MIC (*n* = 32)	Control (*n* = 33)	*P*-value (*T*-test)
Average baseline medication score (points)	2.91 ± 0.52	3.00 ± 0.00	.33
Average 6-month medication score points**)**	2.81 ± 0.85	3.12 ± 0.69	.11
Overall change (points)	−0.10 ± 0.68	+0.12 ± 0.69	.2
Proportion of patients with change, ***n*** (%)	6 (19)	7 (21)	.80^a^

C-MIC, cardio-microcurrent device.

^a^Chi-square test.

### Clinical outcomes by baseline SGLT2 inhibitor use

Despite differences in background medication changes, C-MIC therapy was associated with **similar** treatment effects across all efficacy endpoints, irrespective of baseline SGLT2i use.

For LVEF, the between-group difference in change favouring C-MIC device was +6% (95% CI 3–9; *P* < .001) among patients receiving SGLT2is at baseline and +4% (95% CI 2–7; *P* = .003) among those not receiving SGLT2is. The estimated difference in treatment effect between patients receiving and not receiving SGLT2i was +1% (95% CI −2 to 3; *P* = .500), with no significant treatment-by-SGLT2i interaction (*Figure 1*).

**Figure 1:**
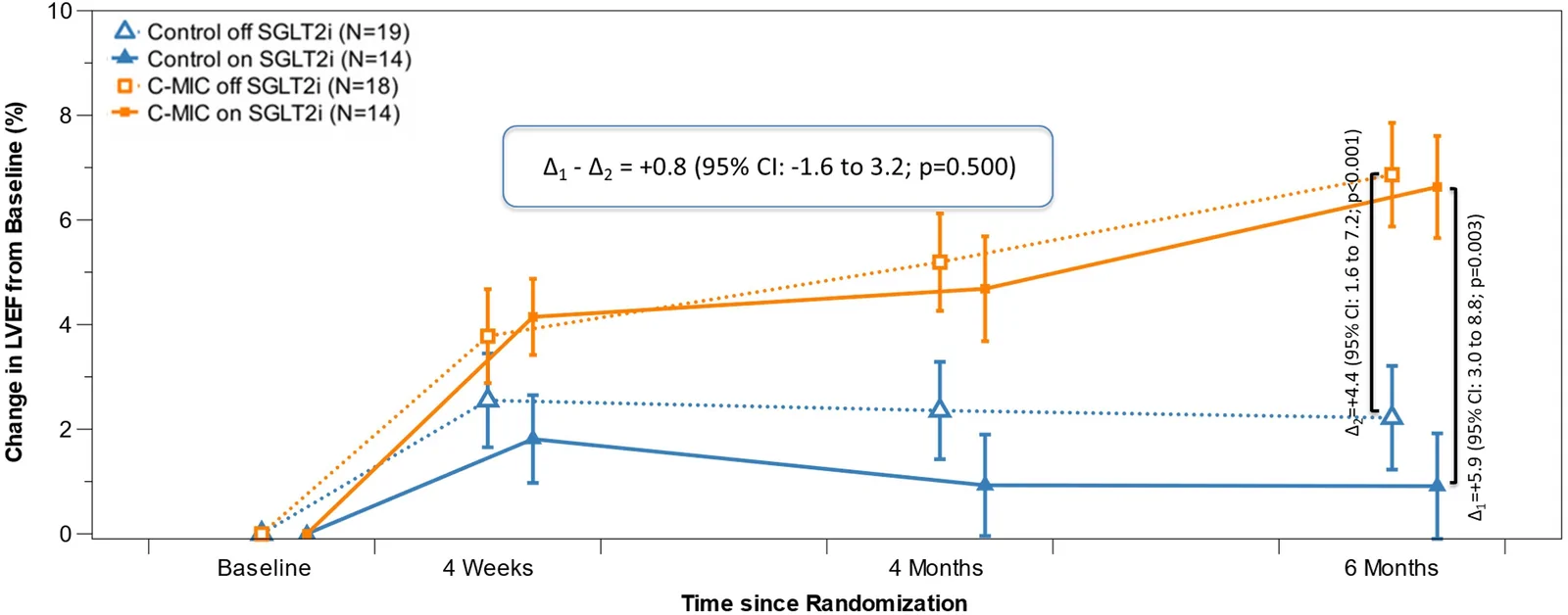
Change in left ventricular ejection fraction (LVEF) at baseline and at various follow-up intervals stratified by baseline sodium-glucose cotransporter-2 inhibitor (SGLT2i) use in the modified intention-to-treat population.

Similarly, improvements in KCCQ-OSS and 6MWD were similar across SGLT2i subgroups. For KCCQ-OSS, the between-group difference in change favouring C-MIC was +42 points (95% CI 26–58; *P* < .001) among patients receiving SGLT2is at baseline and +40 points (95% CI 28–51; *P* < .001) among those not receiving SGLT2is. The estimated difference in treatment effect was −2 (95% CI −16 to 12; *P* = .800) between SGLT2i subgroups (*Figure 2*).

**Figure 2:**
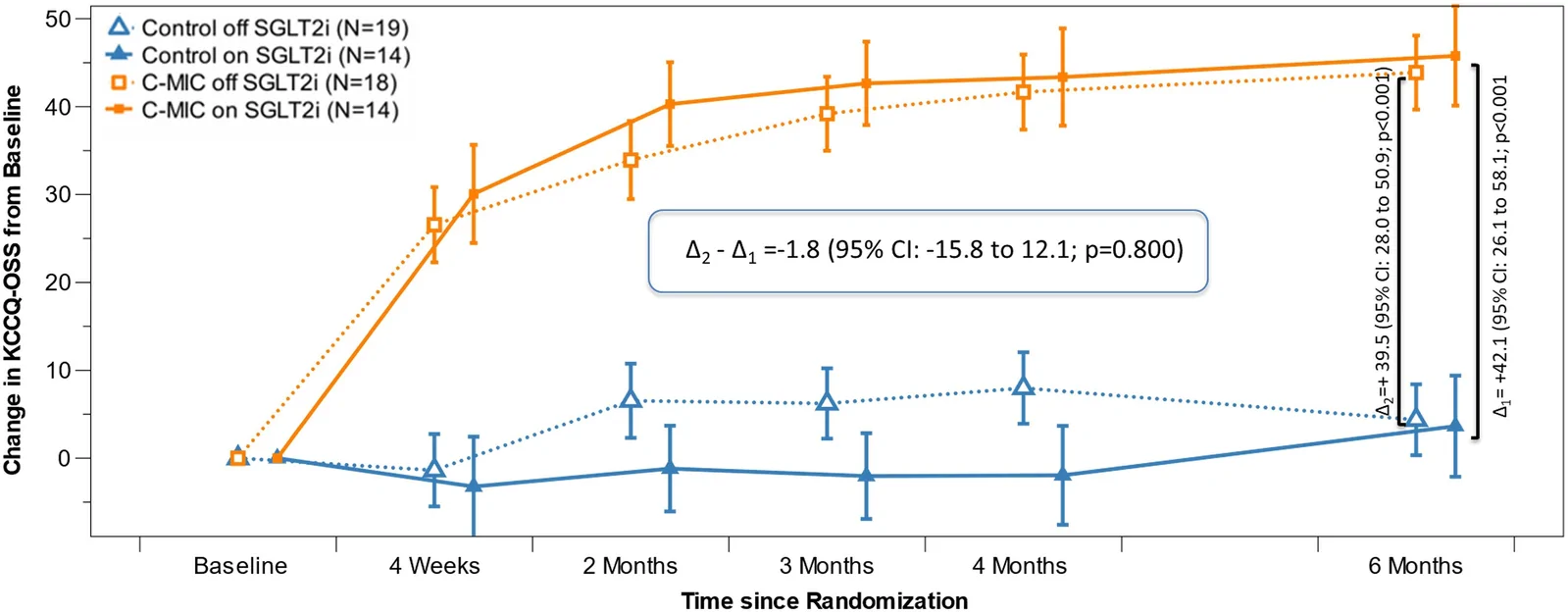
Change in Kansas city cardiomyopathy questionnaire overall summary score (KCCQ-OSS) at baseline and at various follow-up intervals stratified by baseline sodium-glucose cotransporter-2 inhibitor (SGLT2i) use in the modified intention-to-treat population.

For 6MWD, the between-group difference in change favouring C-MIC was +150 metres (95% CI 88–212; *P* < .001) among patients receiving SGLT2is at baseline and +141 metres (95% CI 63–219; *P* < .001) among those not receiving SGLT2is. There was no significant treatment-by-baseline SGLT2i interaction (−16, 95% CI −80 to 48; *P* = .600) (*Figure 3*).

**Figure 3:**
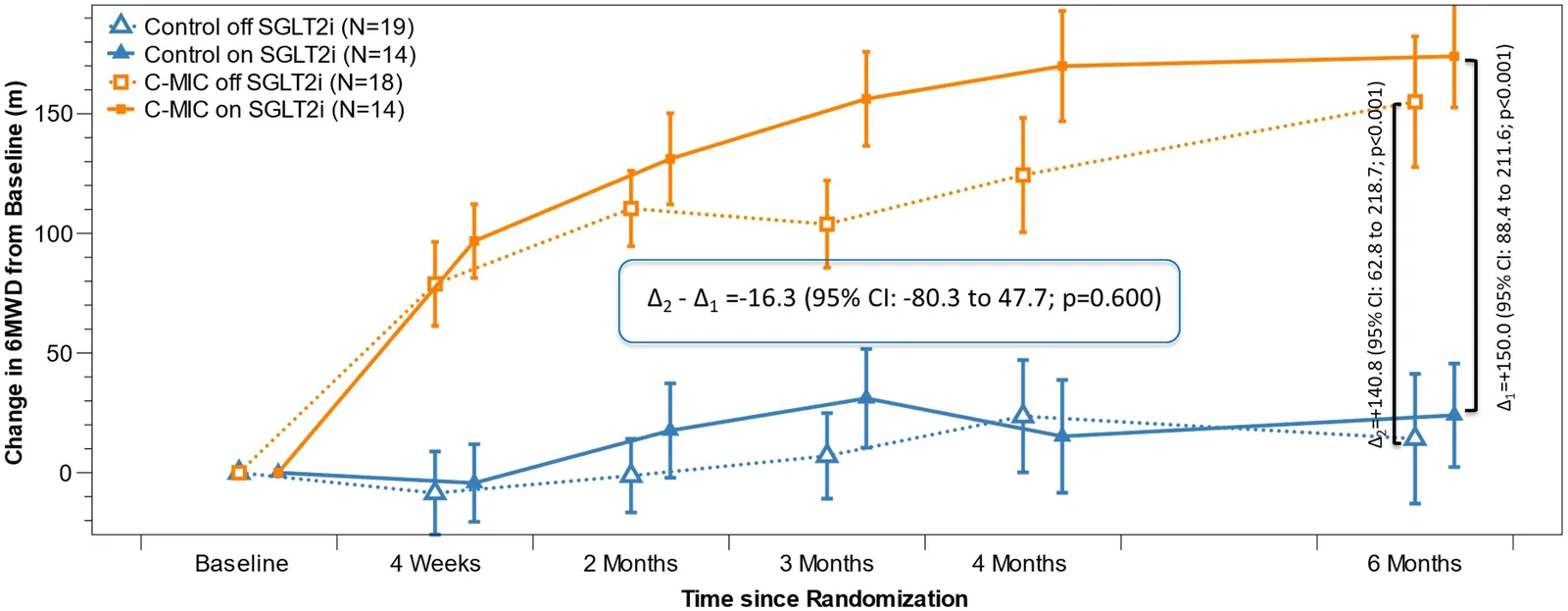
Change in 6-min walk distance (6MWD) at baseline and to various follow-up intervals stratified by baseline sodium-glucose cotransporter-2 inhibitor (SGLT2i) use in the modified intention-to-treat population.

## Discussion

In this secondary analysis of the C-MIC II trial enrolling symptomatic patients with chronic HFrEF (predominantly NYHA class III), background HF medication adjustments occurred with similar frequency in both treatment arms. However, medication intensity increased in control patients, including GDMT and diuretics, while remaining stable or decreasing in patients treated with C-MIC therapy. Despite greater pharmacologic intensification in the control group, clinical outcomes consistently favoured C-MIC therapy. Baseline SGLT2i use was not associated with differences in primary or secondary endpoints (Graphical Abstract). Overall, these findings suggest that the clinical benefits observed with C-MIC therapy cannot be explained by differences in background HF medication use.

The GDMT intensity score offers an objective framework to quantify and compare background HF therapy across study groups and to assess whether novel interventions provide incremental benefit.^12,13^ Background HF therapy remained similar between groups throughout follow-up, with both receiving optimal GDMT. Medication adjustments occurred in both groups during the 6-month study period. Among patients not treated with a C-MIC device, increases in medication intensity were observed, driven primarily by escalation of loop diuretics in addition to GDMT. In contrast, there was a slight decrease in GDMT and diuretic intensity in patients receiving C-MIC therapy. Increases in diuretic intensity may reflect greater requirements for decongestion and have been associated with adverse HF outcomes.^14,15^ However, increases in GDMT intensity may reflect therapeutic optimization in clinically stable patients and should not be interpreted solely as markers of disease progression. Given the exploratory nature of this analysis and limited sample size, these findings should be interpreted as a descriptive summary of medication trajectories and considered hypothesis-generating.

Although the overall use of SGLT2is was modest (43%), this aligns with uptake rates reported in contemporary HF trials conducted during the same period^8^ and likely reflects delayed adoption and limited availability of these drugs in several Eastern European countries during the study period. Given the strong evidence that SGLT2is exert early benefits on symptoms, functional capacity, and cardiac remodelling,^1–6^ our findings argue against differential GDMT intensification as the primary driver of the observed improvements in LVEF and clinical outcomes. Rather, the similarity of treatment effects across SGLT2i subgroups supports a therapeutic effect of C-MIC therapy that appears independent of pharmacologic escalation. Similarly, improvements in patient-reported KCCQ-OSS and 6MWD were comparable between SGLT2i subgroups, despite evidence that SGLT2is alone can produce early gains in these domains.^1,4^

A key consideration in the evaluation of emerging therapies for HFrEF is whether observed benefits are truly incremental to those achieved with established GDMT. In the C-MIC II trial, C-MIC therapy was evaluated in symptomatic ambulatory patients with non-ischaemic chronic HFrEF despite receiving optimal medical therapy.^10^ In the present report, we report that the C-MIC device demonstrates a consistent benefit and improves cardiac function, exercise capacity, and quality of life when added to core GDMT for HFrEF. The demonstration that neither of these agents, including SGLT2is, modified the response to cardiac microcurrent therapy supports the view that the C-MIC system acts in a mechanistically independent and complementary manner to existing pharmacologic therapies for HFrEF. These results are concordant with prior C-MIC studies demonstrating sustained improvements in LVEF and clinical status, including durable benefits and a favourable safety profile observed at 2-year follow-up in first-in-human cohorts.^9,10^ Together, these data support the concept that C-MIC therapy provides incremental benefits beyond optimized GDMT in patients with chronic HFrEF.

The precise biological mechanisms underlying cardiac microcurrent therapy remain incompletely defined, but the proposed pathways include modulation of cardiomyocyte membrane potentials, promotion of tissue repair, anti-inflammatory effects, improvement in contractile synchrony and reduction in myocardial fibrosis and oedema.^16–19^ These effects may collectively contribute to favourable cardiac remodelling and myocardial functional recovery. Ongoing follow-up from the C-MIC II trial will further inform the long-term safety and durability of device implantation at 2 years, clarifying the potential of the C-MIC device as an adjunctive therapy in patients with chronic HFrEF.

### Limitations

This study has several limitations. First, this study is limited by a modest sample size and the exploratory nature of subgroup comparisons. Although no statistically significant treatment-by-baseline SGLT2i interactions were observed across efficacy endpoints, these findings should be interpreted cautiously given the limited statistical power of these subgroup analyses. Second, the predefined semi-quantitative medication scoring system represents a descriptive measure of longitudinal changes in background therapy and should not be interpreted as a direct measure of clinical status or treatment response. The score cannot fully capture nuances in clinical decision-making or the timing of therapy initiation and assumes a linear and equivalent clinical weighting across drug classes and dose modifications, which may not fully reflect their differential clinical effects. Additionally, maximum tolerated doses of individual GDMT classes were not prospectively collected in a standardized manner and could not be reliably assessed retrospectively. Third, changes in background therapy during follow-up may have been influenced by the evolving clinical status of patients, introducing potential time-varying confounding by indication that was not specifically accounted for in the analysis. Fourth, the study was not powered to detect interactions between GDMT changes and treatment effects of C-MIC therapy. Accordingly, these findings should be interpreted as hypothesis-generating and should be validated in larger, adequately powered studies.

## Conclusions

In the C-MIC II trial, HF medication adjustments occurred with similar frequency across treatment groups among symptomatic patients with chronic HFrEF (predominantly NYHA class III). GDMT and diuretic intensity decreased in C-MIC-treated patients while increasing in controls. Improvements in LVEF, 6MWD and KCCQ-OSS scores were consistent regardless of baseline SGLT2i use, suggesting that the observed benefits of C-MIC therapy are unlikely to be driven by differential intensification of background HF pharmacotherapy.

## Declarations

### Data Availability

No data were generated or analysed for this manuscript.

### Pre-registered Clinical Trial Number

None supplied.
